# HO-1 Limits the Efficacy of Vemurafenib/PLX4032 in BRAF^V600E^ Mutated Melanoma Cells Adapted to Physiological Normoxia or Hypoxia

**DOI:** 10.3390/antiox11061171

**Published:** 2022-06-14

**Authors:** Anna Lisa Furfaro, Giulia Loi, Caterina Ivaldo, Mario Passalacqua, Gabriella Pietra, Giovanni Enrico Mann, Mariapaola Nitti

**Affiliations:** 1 Department of Experimental Medicine, University of Genoa, Via L.B. Alberti 2, 16132 Genova, Italy; annalisa.furfaro@unige.it (A.L.F.); giulia.loi@edu.unige.it (G.L.); caterina.ivaldo@edu.unige.it (C.I.); mario.passalacqua@unige.it (M.P.); gabriella.pietra@unige.it (G.P.); 2 Laboratory of Immunology, IRCCS Ospedale Policlinico San Martino, 16132 Genova, Italy; 3 King’s British Heart Foundation Centre of Research Excellence, School of Cardiovascular and Metabolic Medicine & Sciences, King’s College London, 150 Stamford Street, London SE1 9NH, UK; giovanni.mann@kcl.ac.uk

**Keywords:** HO-1, NK ligands, melanoma, oxygen tension, NRF2, physiological normoxia, hypoxia, response and/or resistance to therapy, target therapy

## Abstract

Induction of heme oxygenase 1 (HO-1) favors immune-escape in BRAF^V600^ melanoma cells treated with Vemurafenib/PLX4032 under standard cell culture conditions. However, the oxygen tension under standard culture conditions (~18 kPa O_2_) is significantly higher than the physiological oxygen levels encountered in vivo. In addition, cancer cells in vivo are often modified by hypoxia. In this study, MeOV-1 primary melanoma cells bearing the BRAF^V600E^ mutation, were adapted to either 5 kPa O_2_ (physiological normoxia) or 1 kPa O_2_ (hypoxia) and then exposed to 10 μM PLX4032. PLX4032 abolished ERK phosphorylation, reduced Bach1 expression and increased HO-1 levels independent of pericellular O_2_ tension. Moreover, cell viability was significantly reduced further in cells exposed to PLX4032 plus Tin mesoporphyrin IX, a HO-1 inhibitor. Notably, our findings provide the first evidence that HO-1 inhibition in combination with PLX4032 under physiological oxygen tension and hypoxia restores and increases the expression of the NK ligands ULBP3 and B7H6 compared to cells exposed to PLX4032 alone. Interestingly, although silencing NRF2 prevented PLX4032 induction of HO-1, other NRF2 targeted genes were unaffected, highlighting a pivotal role of HO-1 in melanoma resistance and immune escape. The present findings may enhance translation and highlight the potential of the HO-1 inhibitors in the therapy of BRAF^V600^ melanomas.

## 1. Introduction

Cancer cells take advantage of the over activation of antioxidant defenses to counteract the imbalance in the production of reactive oxygen species (ROS) resulting from changes in cell metabolism [1,2]. Indeed, upregulation of antioxidant defenses enables cancer cells to increase their resistance to therapy and survival, favoring disease progression [3,4].

Heme oxygenase 1 (HO-1), the inducible isoform of heme oxygenases, has been implicated in cancer progression as it seems to enhance aggressiveness and resistance to therapies [5], leading to poor prognosis/outcome. Moreover, HO-1 has recently gained clinical significance as a marker of tumor progression [6]. HO-1 can be activated via different redox sensitive signaling pathways such as NF-kB, AP-1 and NRF2 [7]. In this context, it is well recognized that upregulation of Nuclear factor erythroid 2 p45-related factor (NRF2), a master regulator of cell responses to stressors, increases tumor cell resistance to therapies [8] whilst inhibition of NRF2 increases the efficacy of antitumor therapies to reduce cancer progression [9]. Although NRF2 controls a plethora of genes with antioxidant and detoxifying activity, the specific association between NRF2 and HO-1 activation and their correlation with tumor progression has been well documented in different tumors [10].

Cutaneous melanoma is the most aggressive type of skin cancer characterized by a high ability to metastasize. Although melanoma is the less common skin tumor, it has the higher mortality rate, up to 75% [11]. About 50% of cases harbors the point mutation at codon 600 of BRAF (BRAF^V600^) [12] and the constitutive phosphorylation of ERK pathway favors evasion of apoptosis, invasiveness and immune escape [13]. The development of BRAF^V600^-targeted drugs such as Vemurafenib/PLX4032 efficiently evoke tumor regression, but unfortunately, within a year, the disease relapses with the more aggressive phenotype [14,15]. Importantly, Natural Killer (NK) cell infiltration in melanoma positively correlates with tumor regression, as their production of tumor necrosis factor-α (TNF-α), interleukin 10 (IL-10), CCL2 (MCP-1), CCL3 (MIP1-α) crucially recruits other hematopoietic cells in areas of inflammation favoring immune recognition [16]. However, NK cell recognition can be evaded by melanoma cells generating the less immunogenic and more progressive tumor variant. Thus, a complete understanding of the molecular mechanisms underlying melanoma cell evasion from NK cell recognition is fundamental. Using different primary melanoma cells bearing BRAF^V600^ mutations, we recently established that cell exposure to Vemurafenib/PLX4032 upregulates HO-1 expression that, in turn, reduces drug efficacy, by limiting drug-induced cell death and NK cell dependent recognition and killing [17]. However, our previous study and the majority of studies with cancer cells are conducted under standard culture conditions in which cells are maintained under atmospheric oxygen (O_2_) levels (18 kPa O_2_). This oxygen tension is clearly higher than the actual O_2_ tension in tissues that ranges between 3 and 8 kPa in the skin [18]. Notably, hyperoxic cell culture conditions can influence cellular redox status due to an upregulation of antioxidant responses, thereby limiting the relevance of the results obtained [18].

In addition, in an expanding tumor mass, areas with a reduced availability of O_2_ are generated. In hypoxic condition (less than 1% O_2_ availability) cancer cells not only increase their angiogenetic capability, but also modify their response to drugs [19]. Moreover, the possibility to elude immune surveillance is increased as demonstrated in different tumors [20] and in melanoma as well [21].

Thus, to increase the clinical relevance of results obtained in vitro on redox-regulated parameters, it seems fundamental that experiments are conducted with cells adapted long-term to physiological normoxia and, in the case of cancer cells, adapted to hypoxia. In this study, we adapted primary melanoma cells bearing the BRAF^V600E^ mutation to normoxia or hypoxia and demonstrated that induction of HO-1 limits the efficacy of Vemurafenib/PLX4032 in tumor regression. Furthermore, we established that inhibition of HO-1 activity significantly improves the efficacy of PLX4032, highlighting the clinical potential of HO-1 inhibitors to improve targeted therapy in melanoma.

## 2. Materials and Methods

### 2.1. Culture and Adaptation of MeOV-1 Cells under Defined O_2_ Tensions

A primary BRAF^V600E^ MeOV-1 melanoma cell line (MeOV-1), derived from a metastatic lesion, was obtained with consent procedures (nOMA09.001) approved by the International Ethics Board of the National Cancer Institute (IRCCS Policlinico San Martino Hospital, Genoa, Italy) and authenticated by STR profile (Cell ID^TM^ system, Promega, Milan, Italy).

MeOV-1 cells were cultured in RPMI 1640 medium (Euroclone, Milan, Italy) supplemented with 10% fetal bovine serum (Euroclone), 2 mM glutamine (Sigma-Aldrich, Milan, Italy) and 1% penicillin/streptomycin (Sigma-Aldrich) and sub-cultured every 5 days at 1:5 under standard cell culture conditions gassed with room air and 5% CO_2_.

Cells were adapted to lower O_2_ tensions by culturing cells for 5 days at 37 °C in an O_2_-regulated dual workstation (Scitive, Baker-Ruskinn, Sanford, ME, USA) under 5% CO_2_ humid atmosphere and physiological normoxia (5 kPa) or hypoxia (1 kPa) to ensure adaptation of the redox proteome [18]. Cells were maintained in the O_2_-regulated workstation with all sub-culture, treatments and extraction of cell lysates conducted within the workstation to avoid re-exposure of cells to room air.

### 2.2. Treatment of Primary Melanoma Cells

Cells were treated with 10 μM PLX4032 (Selleckchem, Houston, TX, USA) for different times. In some experiments, cells were co-treated for 24 h with 10 μM Tin mesoporphyrin IX (HO-1 inhibitor, SnMP, Cayman Chemical Company, Ann Arbor, MI, USA) and 10 μM PLX4032. The doses were selected based on our previous work [17]. Diethylmaleate (DEM, 100 µM) was used as a stressor known to lead to NRF2 activation [22]. DMSO was used to dissolve PLX4032, SnMP and DEM.

### 2.3. MTT Assay

Cell viability was evaluated by using 3-(4,5-dimethyl-thiazol-2-yl)-2,5-diphenyltetrazolium bromide (MTT, Sigma-Aldrich) assay. At the end of the treatments, cells were incubated with MTT solution (10% of 5 mg/mL MTT stock solution) in serum-free medium without phenol red for 3 h at 37 °C and the insoluble formazan salts dissolved in pure DMSO (Sigma-Aldrich). The absorbance at 570 nm was measured in a spectrophotometric plate reader (CLARIOstar, BMG Labtech, Aylesbury, UK) or iMark microplate reader (Bio-Rad, Milan, Italy). Mean values for each treatment were calculated and expressed as a percentage relative to the untreated cells.

### 2.4. Small Interfering RNA

Small interfering RNA was performed by using a specific pool of oligonucleotides against human NRF2 (On-TargetPlus SMART pool nuclear factor (erytroid-derived 2)-like 2; Dharmacon, Lafayette, CO, USA). MeOV-1 cells were transfected with 120 pmoles of siRNA (siNRF2) for 24 h using Lipofectamine 2000 (Life Technologies, Carlsbad, CA, USA) according to the manufacturer’s instructions. Cells were then exposed to 10 µM PLX4032 or 100 µM DEM for 24 h. The efficiency of silencing was verified monitoring NRF2 mRNA levels and the specificity by using a non-targeting pool (NoT) of oligonucleotides (On-TargetPlus siControl non targeting pool; Dharmacon) at the same concentration used for siNRF2.

### 2.5. RNA Extraction, Reverse Transcriptase and Polymerase Chain Reaction

Total RNA was extracted using TriZol reagent (ThermoFischer, Waltham, MA, USA) or RNeasy^®^ Mini Kit (Qiagen GmbH, Hilden, Germany) following the manufacturer’s instructions and quantified by using a NanoDrop™ spectrophotometer (ThermoFischer). An amount of 500 ng of total RNA was reverse transcribed into cDNA by using random hexamer primers (Tib MolBioL, Genoa, Italy) and SuperScriptTM II Reverse Transcriptase (Life Technologies).

For semi-quantitative PCR, cDNA was amplified (Mastercycler, Eppendorf, Milan, Italy) using Platinum Taq Polymerase (Life Technologies) and specific primers for HO-1, GCLC, GCLM, NQO1 and NRF2 (Tib MolBiol, Genoa, Italy). 18S ribosomal RNA (18S) was used as a housekeeping gene. PCR products were separated by electrophoresis on 2% agarose gel pre-stained with ethidium bromide, visualized under ultraviolet light, and quantified by densitometric analysis using a specific software (GelDoc; Bio-Rad, Hercules, CA, USA).

For quantitative PCR (qPCR), diluted cDNA was amplified (LyghtCycler 96 SW, Roche, Basel, Switzerland) using Luna Universal qPCR Master Mix (Euroclone) and specific primers for B7H6 and ULBP3. Samples were pre-incubated at 95 °C for 120 s, followed by 40 amplification cycles of 95 °C for 10 s, 59 °C for 30 s and 68 °C for 20 s. Gene relative expression was calculated with the 2^−ΔΔCT^ methods using β-actin as internal reference. Results were expressed as percentage of untreated samples.

Primers sequences used are listed in Table 1.

### 2.6. Total Protein Extraction

At the end of treatments, cells were lysed in RIPA buffer as previously described [23]. Protein content was measured using BCA assay (Pierce, ThermoScientific).

### 2.7. Immunoblot Analysis

Protein cell lysates were denaturated in Laemmli buffer, subjected to SDS-polyacrylamide gel electrophoresis (200 Volt for 50 min) using 4–20% Mini-Protean TGX ™ Gels precast (Bio-Rad). After blotting, PVDF membranes (GE Healthcare, Amersham Place, UK) were blocked in non-fat dry milk (5% *w*/*v* in 1X tTBS) and then incubated with antibodies against ERK, p-ERK (1:1000, rabbit polyclonal antibody, Cell Signaling Technology, Leiden, The Netherlands), HO-1 (1:2000, rabbit polyclonal antibody, ORIGENE, Herford, Germany), Bach1 (1:4000, rabbit polyclonal antibody, Bethyl Lab, Montgomery, TX, USA), and HIF-1α (1:1000, rabbit polyclonal antibody, Abcam, Cambridge, UK). Bands were detected by enhanced chemiluminescence (GE Healthcare) after incubation with specific secondary antibodies (1:10,000, anti-rabbit and anti-mouse, GE Healthcare). Membranes were stripped and re-probed with anti-actin (1:10,000, mouse monoclonal antibody, Sigma-Aldrich). Developed films were quantified by densitometric analysis using a GelDoc software (Bio-Rad).

### 2.8. Immunofluorescence

MeOV-1 cells were seeded into 8-well chamber slides at 150,000 cells per well and treated with 10 μM PLX4032 or 100 µM DEM for 1–2 h. At the end of treatments, cells were fixed and permeabilized with cold 100% methanol and incubated with anti NRF2 antibody (1:100, rabbit polyclonal C-20, Santa Cruz, CA, USA) at 4 °C overnight. NRF2 expression was detected using anti-rabbit ALEXA 488 (1:500, ThermoFisher). Nuclei were stained with TO-PRO-3 (1:1000, ThermoFisher). Images were collected using a three-channel TCS SP2 laser scanning confocal microscope (Leica Microsystems, Wetzlar, Germany).

### 2.9. Statistical Analyses

Data are expressed as mean ± S.E.M. of measurements in n = 3–5 independent experiments and analyzed using One-way analysis of variance (ANOVA) or Dunnett’s multiple comparison tests when comparing more than three mean values. A Student’s *t*-test was used when appropriate as indicated in figure legends.

## 3. Results

### 3.1. Exposure to PLX4032 Abolished ERK Phosphorylation, Downregulated Bach1 and Upregulated HO-1 in MeOV-1 Cells Adapted to Normoxia or Hypoxia

In order to investigate whether the reduction of oxygen tension could modify BRAF^V600E^ melanoma response to PLX4032, MeOV-1 cells were adapted for 5 days to normoxia (5 kPa O_2_) or hypoxia (1 kPa O_2_) and then exposed to 10 µM PLX4032 for 24 h.

**Figure 1:**
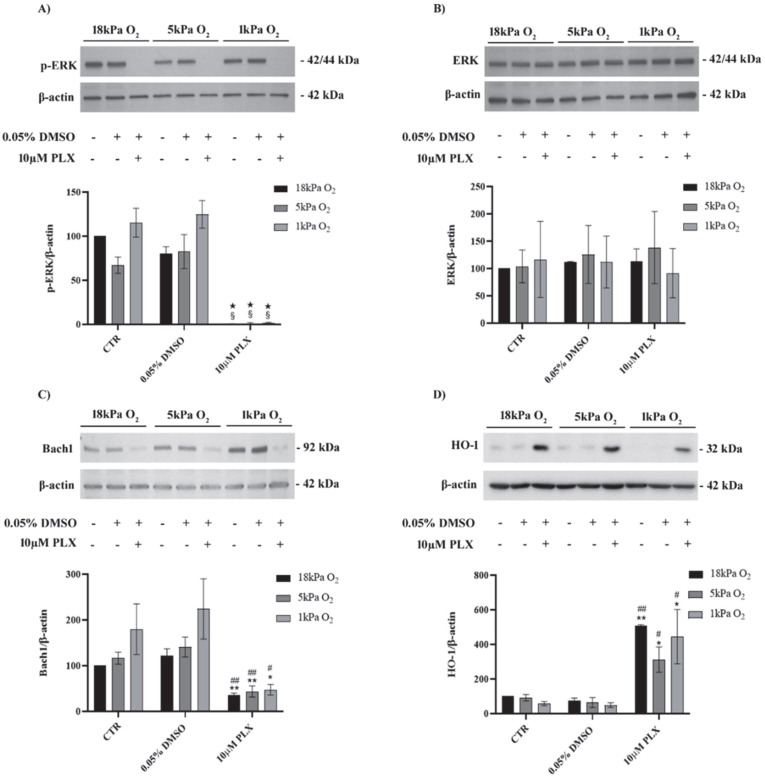
Immunoblot analysis of pERK (**A**), total ERK (**B**), Bach1 (**C**) and HO-1 (**D**). MeOV-1 cells were adapted for 5 days to 18 kPa, 5 kPa or 1 kPa O_2_ and then treated with 10 µM PLX4032 (or 0.05% DMSO) for 24 h. β-actin was used as loading control. Immunoblots are representative of 3–4 independent experiments. Data denote mean ± S.E.M., n = 3–4 independent cell cultures, § *p* < 0.001 vs. CTR; ★ *p* < 0.001 vs. DMSO; ★★ *p* < 0.01 vs. CTR; # *p* < 0.05 vs. DMSO; ## *p* < 0.01 vs. DMSO.

As shown in Figure 1, PLX4032 abolished ERK phosphorylation in cells adapted to 5 kPa O_2_ or 1 kPa O_2_ with negligible differences compared to cells cultured under standard, hyperoxic (18 kPa O_2_) conditions (Figure 1A). No changes were observed in the expression level of total ERK (Figure 1B). Moreover, expression of Bach1 was reduced and HO-1 increased after 24 h exposure to PLX4032 in all oxygen conditions (Figure 1C,D).

### 3.2. HO-1 Inhibition Improves Efficacy of PLX4032 in Reducing MeOV-1 Cell Viability under Different Oxygen Tensions

**Figure 2:**
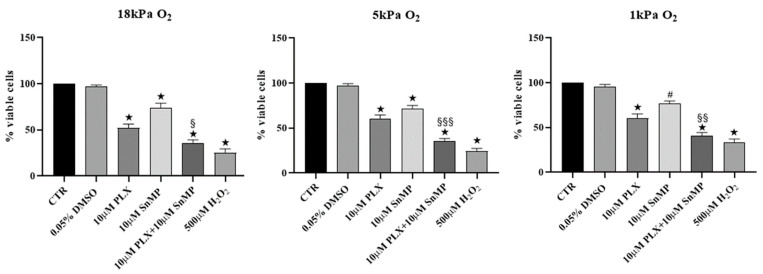
Melanoma cell viability after PLX4032 treatment and HO-1 inhibition. Cell viability was determined using the MTT assay in MeOV-1 cells seeded into 96-well plates and adapted for 5 days to 18 kPa O_2_, 5 kPa or 1 kPa O_2_. Cells were then treated with 10 µM PLX4032, 10 µM SnMP and combined treatment for 24 h. Data denote mean ± S.E.M., n = 5 independent cell cultures, ★ *p* < 0.005 vs. respective CTR and DMSO; # *p* < 0.05 vs. respective CTR and DMSO; § *p* < 0.05 vs. respective PLX4032; §§ *p* < 0.005 vs. respective PLX4032; §§§ *p* < 0.001 vs. respective PLX4032.

Our previous studies highlighted a key role of HO-1 in limiting the efficacy of PLX4032 on BRAF mutated melanoma cells [17] cultured under 18 kPa O_2_. Now, we demonstrate that exposure of MeOV-1 cells to PLX4032 decreased cell viability of about 45% in cell cultured not only at 18 kPa O_2_, but also at 5 kPa O_2_ and 1 kPa O_2_. Importantly, co-treatment with PLX4032 and 10 µM SnMP, a competitive inhibitor of HO-1, efficacy further reduced cell viability in comparison to cells exposed to PLX4032 alone under all O_2_ tensions tested (Figure 2).

### 3.3. HIF-1a Is Expressed in Untreated BRAF^V600E^ MeOV-1 and Reduced by PLX4032

**Figure 3:**
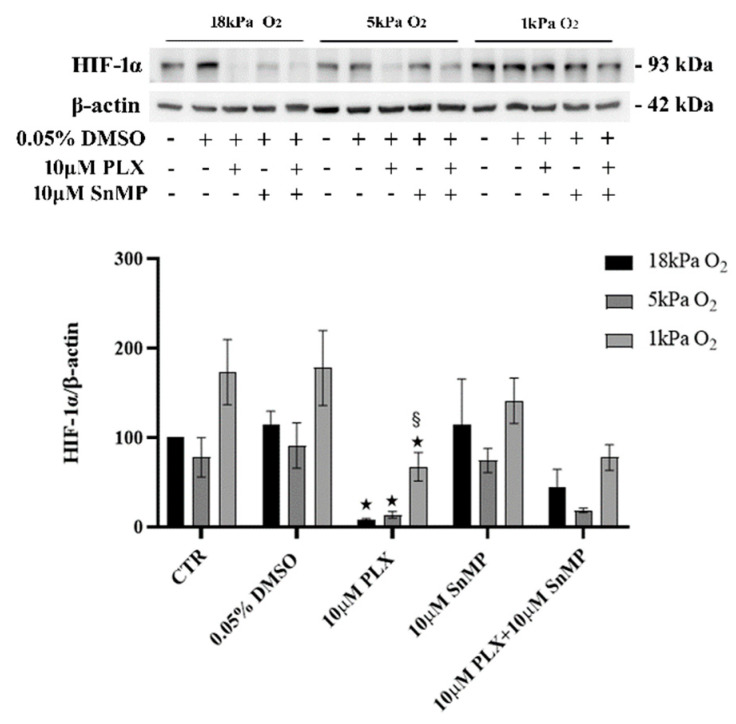
HIF-1α protein levels in MeOV-1 cells adapted for 5 days to different O_2_ levels. Immunoblot analysis of HIF-1α protein expression in MeOV-1 cells cultured for 5 days under 18 kPa O_2_, 5 kPa O_2_ or 1 kPa O_2_ and then treated with 10 µM PLX4032 (or 0.05% DMSO) and 10 µM SnMP for 24 h under the same O_2_ levels. β-actin was used as a loading control. Immunoblots are representative of 3 independent MeOV-1 cultures. Data denote mean ± S.E.M. ★ *p* < 0.05 vs. respective CTR and DMSO; § *p* < 0.005 vs. PLX under 18 kPa and PLX under 5 kPa O_2_.

As shown in Figure 3, immunoblot analysis revealed constitutive expression of HIF-1α under basal conditions with no modification detected in control cells maintained long-term under physiological normoxia (5 kPa O_2_). A trend for increased stabilization of HIF-1α can be observed only in control cells adapted to hypoxia. Moreover, HIF-1α protein levels were significantly reduced by PLX4032 at 18 kPa, 5 kPa O_2_ and 1 kPa O_2_ compared with their respective controls. HO-1 inhibition had no effect on the modification of HIF-1α levels following the exposure to PLX4032 under different O_2_ levels.

### 3.4. HO-1 Inhibition Favors Expression of NK Ligands in MeOV-1 Cells Exposed to PLX4032 Independent of Oxygen Tension

**Figure 4:**
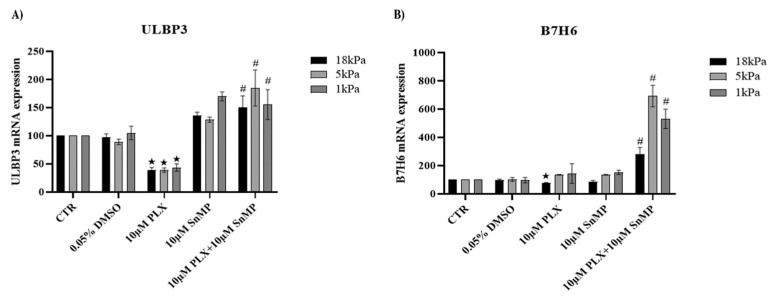
mRNA expression of ULBP3 and B7H6 in MeOV-1 cells co-treated with PLX4032 and SnMP. ULBP3 (**A**) and B7H6 (**B**) mRNA expression was assessed by qPCR in MeOV-1 cells cultured under standard culture conditions (18 kPa O_2_) or adapted for 5 days to 5 kPa or 1 kPa O_2_. Cells were then exposed to 10 μM PLX4032 ± 10 μM SnMP for 24 h. Data shown are representative of 2 or 3 independent experiments for each experimental condition. Date denote mean ± S.E.M., ★ *p* < 0.05 vs. CTR and DMSO; # *p* < 0.05 vs. PLX4032.

qPCR analysis confirmed that PLX4032 reduced the expression of the NK ligands ULBP3 and B7H6 in cells cultured under standard 18 kPa O_2_ conditions. Importantly, adaptation to 5 kPa O_2_ or 1 kPa O_2_ did not modify this effect as far as ULBP3 is concerned. HO-1 inhibition always restored ULBP3 expression (Figure 4A). In contrast, B7H6 mRNA expression was not downregulated by PLX4032 in cells adapted to normoxia or hypoxia. HO-1 inhibition was able to strongly increase B7H6 mRNA expression (Figure 4B).

### 3.5. PLX4032 Selectively Upregulates NRF2-Dependent HO-1 Expression

**Figure 5:**
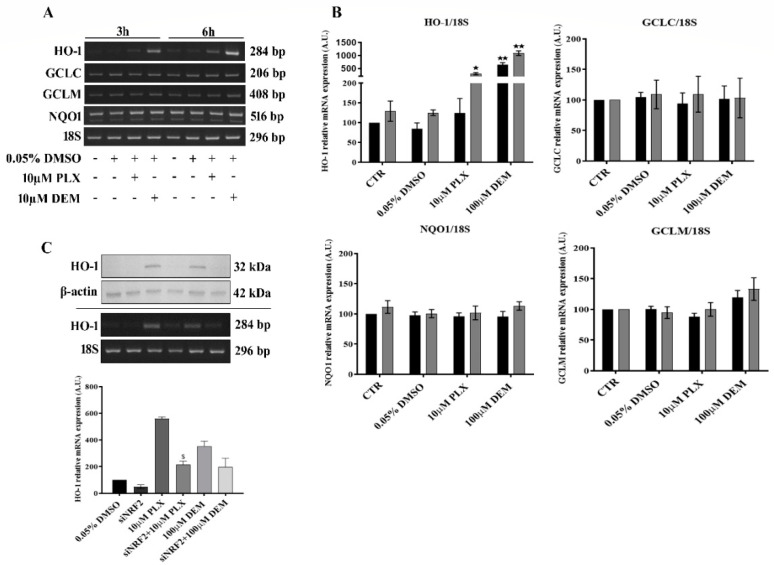
PLX4032 induces NRF2-dependent HO-1 expression. HO-1, GCLC, NQO1 and GCLM mRNA expression was assessed by RT-PCR in MeOV-1 cells treated with 10 μM PLX4032 for 3 h and 6 h. Diethylmaleate (100 μM DEM) was used as a positive control and 18S mRNA was used as a housekeeping gene. Representative blots of 3 independent experiments are shown in (**A**). Densitometric analysis for each gene shown in (**B**) where data denote mean ± S.E.M. ★ *p* < 0.05 vs. respective CTR; ★★ *p* < 0.001 vs. CTR. The results of NRF2 silencing on HO-1 expression are shown in (**C**): HO-1 protein (upper) and mRNA (lower) expression were assessed by immunoblotting and by RT-PCR, using DEM as a positive control. Actin protein level and 18S mRNA were used as internal controls, respectively. Representative blots of 3 independent experiments are shown, with densitometric analyses of HO-1 mRNA expression. Data denote mean ± S.E.M. s *p* < 0.005 vs. PLX.

RT-PCR analyses revealed that after 24 h of PLX4032 treatment, HO-1 expression was upregulated while GCLM, GCLC and NQO1, whose expression is also controlled by NRF2, were not modified (Supplementary Figure S2). Moreover, short time analysis at 3 and 6 h showed HO-1 upregulation in response to PLX4032 and no changes of GCLM GCLC or NQO1 (Figure 5A,B). Furthermore, by mean of immunofluorescence, nuclear localization of NRF2 was observed in MeOV-1 exposed to PLX4032 for 1 h and 2 h (Supplementary Figure S3). In addition, siRNA for NRF2 prevented protein overexpression and reduced mRNA overexpression of HO-1 due to PLX4032 exposure (Figure 5C), proving that PLX4032 induced HO-1 expression through an early translocation of NRF2.

## 4. Discussion

In the present study, upregulation of heme oxygenase 1 (HO-1) was shown for the first time in primary BRAF^V600E^ melanoma cells adapted to physiological oxygen tension and hypoxia to limit the efficacy of Vemurafenib/PLX4032. Our approach and findings may be useful to facilitate high throughput studies of HO-1 inhibitors to use in cancer therapies.

Even with significant improvements achieved using targeted therapeutic approaches, melanoma still has a highly unfavorable prognosis. Indeed, 50% of cases are characterized by the continuous activation of the MAPK pathway due to the gain of somatic mutation of BRAF in codon 600 (BRAF^V600^), and therefore can be treated with specific BRAF inhibitors (BRAFi), such as Vemurafenib/PLX4032 with proven efficacy in extending patient survival. Yet, a more aggressive form of the disease often relapses within 1 year [24].

Furthermore, the reduction of antioxidant defenses has been proposed as anticancer, sensitizer therapy in the treatment of melanoma, as recently reviewed [25]. However, also to be considered is the role played by NRF2-dependent antioxidant responses in limiting the side effect of chemotherapeutic drugs on normal tissues, as demonstrated for other tumors [26].

Thus, identifying new potential candidates to improve the efficacy of targeted therapies is pivotal, and the present study strengthens the translational potential of HO-1 inhibitors to improve PLX4032 efficacy both in terms of reduction of tumor cell viability and improvement of immune recognition.

HO-1 acts as a powerful stress sensor in cells, and efficiently increases cell resistance through the activity of its metabolites [27,28]. Indeed, from the catabolism of heme groups, HO-1 leads to the generation of CO and bilirubin which exhibit antioxidant, antiapoptotic and immune-regulatory properties [29]. Thus, in the context of cancer progression, the upregulation of HO-1 can provide cells with resistance to anticancer therapies and has been proposed as an unfavorable prognostic factor in different types of tumors [5,6,10]. Moreover, the role of HO-1 in the progression of melanoma has been highlighted [30].

In a previous study, using primary BRAF^V600^ mutated melanoma cell lines isolated in house from patients, we observed that both silencing and pharmacological inhibition of HO-1 in combination with PLX4032 further reduced cell viability compared to the effect of PLX4032 alone [17]. Importantly, we demonstrated that this effect was not cell-specific but shared by different cell lines expressing BRAF^V600E^ or BRAF^V600D^ mutations. Furthermore, we pointed out that PLX4032 treatment downregulated the expression of the NK cell ligands B7H6 and ULBP3 specifically expressed by the lines we used [31,32], and that HO-1 inhibition was able to efficiently restore NK ligand expression [17].

However, the clinical relevance of findings obtained in vitro may be limited by the high oxygen tension (18 kPa O_2_) that cells encountered under standard cell culture conditions, noting that physiological O_2_ tensions in vivo are significantly lower [18]. Notably, hyperoxia increases the expression of antioxidant enzymes in endothelial cells [33], lung epithelial cells [34] and stem cells [35,36], highlighting the importance of maintaining physiological normoxia in cell culture to improve translation of in vitro findings to animals models and the clinic, as also recently recommended in the context of redox biology [37] and tumor biology [38].

In addition, reduced O_2_ levels in the inner zone of tumor mass work as a driving force in the gain of cell modifications that favor cancer progression [39] and hyperoxia cell culture standard conditions do not recapitulate in in vivo conditions. Thus, by culturing cells in an O_2_-regulated workstation, we were able to adapt primary BRAF^V600E^ MeOV-1 cells long-term (5 days) to physiological normoxia (5 kPa O_2_) or hypoxia (1 kPa O_2_). In our workstation, cells can be maintained long-term under defined O_2_ tensions, with medium equilibration at the given O_2_ tension before use, and with all the experiments performed within the workstation to avoid any fluctuations in ambient O_2_ tension. Even though MeOV-1 cells showed a basal expression of HIF-1α, as discussed later, the efficacy of adaptation was confirmed by its further stabilization observed only under hypoxia, confirming our previous findings in peripheral and brain endothelial cells [33,40,41]. Moreover, under lower O_2_ levels, MeOV-1 cells exhibited a slower growth rate and a modest change of morphology (data not shown), but the efficacy of PLX4032 was not modified.

In experimental conditions similar to ours, using different BRAF^V600E^-mutated melanoma cells, other authors showed that in some cell lines cultured under hypoxia HIF-1α expression was reduced in response to PLX4032 but not in others, even though pERK resulted always reduced [42]. Other authors observed the same efficacy in inhibiting pERK both in normoxia and in hypoxia but pointed out different cell responses to PLX4032, such as an increase cell survival in hypoxia, and hypothesized a by-pass mechanism of cell resistance [43]. We observed a basal overexpression of HIF-1α in MeOV-1 cells, in agreement with other reports for BRAF^V600E^ bearing melanoma, as a results of MAPK over activation [44], and no differences were detected in response to PLX4032 treatment. Indeed, pERK was always completely abolished, HO-1 upregulated, and viability was reduced in cells adapted at 5 kPa O_2_ or 1 kPa O_2_ as well as in cells cultured under standard hyperoxic cell culture conditions (18 kPa O_2_). Importantly, the reduction of cell viability was always further improved by HO-1 inhibition obtained using SnMP.

The differential effects of hypoxia on responses to PLX4032 may be due to the heterogeneity of melanoma cell types, and for this reason, a well-controlled experimental setting seems to be crucial to the translation of findings in vivo.

Furthermore, in order to detail the molecular mechanisms involved in HO-1 upregulation in cells exposed to PLX4032, factors involved in HO-1 induction were investigated. We demonstrated that PLX4032 decreased Bach1 expression, a negative regulator of HO-1 transcription [45,46], in MeOV-1 cells adapted to hyperoxia, normoxia or hypoxia. However, there was a trend for Bach1 protein levels to increase in cells under hypoxia as observed previously in endothelial cells [33]. Thus, Bach1 degradation seems the main molecular mechanism involved in HO-1 induction in our model, since, as also reported by others, the degradation of Bach1 specifically contributes to HO-1 induction [47]. In addition, by silencing NRF2, we showed that HO-1 induction also involved NRF2 activation and nuclear translocation. However, other NRF2-dependent genes, such as GCLM, GCLC and NQO1, were not upregulated under the same experimental conditions. Consistently, we already reported a selective upregulation of HO-1 after NRF2 activation in other experimental conditions [48], highlighting a high degree of complexity in the regulation of NRF2-dependent gene transcription. However, the role played by Bach1 deserves more investigation.

We also analyzed whether cell adaptation to normoxia or hypoxia could interfere with melanoma recognition by NK cells, and the possible involvement of HO-1 induction due to cell exposure to PLX4032. Tumor growth and metastasis are controlled by NK cells that are particularly involved in patrolling melanoma tumor mass preventing growth and diffusion. Indeed, the reduction of NK infiltration and cytotoxic activity correlates with relapse and progression of diseases as shown in BRAFi resistant patients [49]. Thus, overcoming tumor immune escape and restoring NK cell recognition and killing activity remains one of the main therapeutic challenges. Among MeOV-1 cell ligands activating NK cells are B7H6 which binds NKp30, and ULBP3 which binds NKG2D [17,32]. We established that cell exposure to PLX4032 under both normoxia and hypoxia downregulated ULBP3 expression and that HO-1 inhibition by SnMP efficiently restored it. Notably, in cells adapted to normoxia or hypoxia, treatment with PLX4032 did not reduce B7H6 expression but, interestingly, the inhibition of HO-1 was further able to increase the expression of this NK ligand significantly.

To the best of our knowledge, this is the first evidence demonstrating that NK ligand expression is modulated by the BRAF inhibitor PLX4032, and that expression can be restored/upregulated by inhibition of HO-1 in melanoma cells adapted to normoxia or hypoxia. Our findings thus strengthen the potential clinical use of HO-1 inhibitors in preventing or reducing melanoma progression in combination with targeted therapy.

Evidence of HO-1 involvement in immune recognition has been provided in other contexts. HO-1 is involved in the regulation of programmed death-1 ligand 1 (PD-L1) expression in renal cancer cells, while its induction contributes to immune-escape [50]. Moreover, HO-1 inhibition by SnMP improves T-cell specific function in the treatment of patients with Wilms disease [51] and increases immune cell responses in acute myeloid leukemia [52]. Furthermore, HO-1 induction in melanoma has been proved to mediate melanoma progression in obese mice increasing the resistance to T-cell mediated apoptosis [53]. Consistently, in the same model, or in cells exposed to fructose, melanoma cells increased HO-1 expression through the degradation of Bach1 and the induction of NRF2 [54]. In both these works, the inhibition of HO-1 obtained both in vitro and in vivo with OB24, a new imidazole-based HO-1 inhibitor, significantly improved the efficacy of immunotherapy. Importantly, it has been recently shown that specific upregulation of HO-1 in tumor associated macrophages shapes the immunosuppressive tumor microenvironment [55], favoring melanoma progression, whilst inhibition of HO-1 using ZnPPIX efficiently restores immune recognition [56]. However, contrasting results on antitumor activity of immune cells overexpressing HO-1 have been also reported [57].

In our work SnMP, already used clinically to treat hyperbilirubinemic disease [58], has been used as has been proposed for the therapy of breast cancer [59] and melanoma [60]. We have now confirmed the efficacy of this inhibitor in MeOV-1 cells under physiological normoxia and hypoxia increasing its potential clinical translation. Due to the high heterogeneity of cancer cells and the sensitivity of the NRF2-dependent pathway to the oxygen tension, as recently highlighted [37,38], we recommend that carefully defined oxygen tension in the culture of cancer cells be considered particularly in the context of therapeutic strategies targeting NRF2 pathways in order to increase their reproducibility and translatability.

It is important to underline that our findings are derived from the analysis of only one tumor line. Even though MeOV-1 are primary cells derived from a patient, and we have previously shown they behave similarly to other cells bearing BRAF^V600^ mutation [17], the use of only one line is a limitation of the work, also considering the heterogeneity of melanomas. Thus, confirmation using other cell lines is needed.

In summary, our study suggests that pharmacological inhibition of HO-1 can efficiently improve the effect of targeted therapy against BRAF^V600^ mutated melanoma, both by favoring drug-induced cancer cell death and by restoring drug-impaired NK recognition. Furthermore, we demonstrated for the first time, using an experimental setting that closely mimics in vivo conditions and an HO-1 inhibitor already in clinical use, that inhibition of HO-1 might enhance the efficacy of Vemurafenib/PLX4032 as a therapeutic in the treatment of melanoma.

## Figures and Tables

**Table 1:** Primers used for PCR and qPCR.

Gene and ID Number	Primer Forward 5’→3’	Primer Reverse 5’→3’
B7H6 NM_001202439	GAC CTG GAG CCA TTG TGT CT	AAG CTG GAC TGT TCC CTG TG
GCLC NM_001498.4	ATG GAG GTG CAA TTA ACA GAC	ACT GCA TTG CCA CCT TTG CA
GCLM NM_002061.4	CCA GAT GTC TTG GAA TGC	TGC AGT CAA ATC TGG TGG
HO-1 NM_002133.3	TCC TGG CTC AGC CTC AAA TG	CGT TAA ACA CCT CCC TCC CC
NQO1 NM_000903.3	CAC TGA TCG TAC TGG CTC A	GCA GAA TGC CAC TCT GAA T
NRF2 NM_006164.5	CGG TAT GCA ACA GGA CAT TG	ACT GGT TGG GGT CTT CTG TG
ULBP3 NM_024518.3	GCC TCG CGA TTC TTC CGT A	CTG CTC TTC TAG GTG ACC C
β-actin NM_001101.5	CTC CTT AAT GTC ACG CAC GAT TTC	ACA ATG AGC TGC GTG TGG CT
18S NR_145820.1	GGG GCC CGA AGC GTT TAC T	GGT CGG AAC TAC GAC GGT ATC

## Data Availability

All materials, data, and protocols associated with this work are available to readers under the responsibility of the corresponding author M. Nitti.

## Supplementary Materials

The following supporting information can be downloaded at: https://www.mdpi.com/article/10.3390/antiox11061171/s1, Figure S1: NRF2 mRNA expression after silencing and PLX treatment; Figure S2: NRF2 dependent gene analyses after 24 h cell treatment with PLX4032; Figure S3: NRF2 nuclear translocation after acute PLX4032 treatment.
