# Supplementary material for: HO-1 Limits the Efficacy of Vemurafenib/PLX4032 in BRAFV600E Mutated Melanoma Cells Adapted to Physiological Normoxia or Hypoxia
Source: Antioxidants (Basel). 2022 Jun 14;11(6):1171. doi: 10.3390/antiox11061171 (PMC9219655; doi:10.3390/antiox11061171)
Supplement: Supplementary file 1 [file antioxidants-11-01171-s001.zip › antioxidants-1752863-supplementary.pdf]

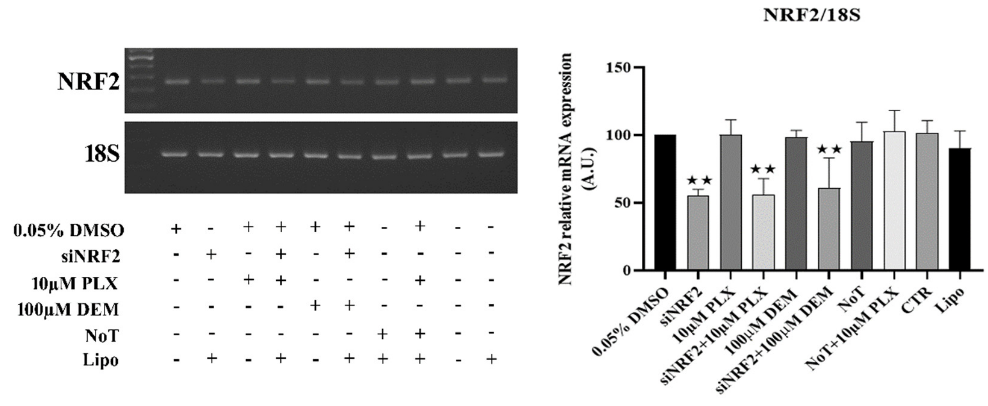

Supplementary Figure S1: NRF2 mRNA expression after silencing and PLX treatment. NRF2 silencing was performed for 24h and cells were then treated with 10μM PLX4032 or 100μM diethylmaleate (DEM). Untreated cells (CTR), exposed to vehicle alone (DMSO), treated with lipophilic Lipofectamine (LIPO) or transfected with a non-targeting oligonucleotides pool (NoT) served as internal controls. 18S mRNA was used as a housekeeping gene. Bands are representative of 3 independent experiments. Data denote mean ± S.E.M., ★★P<0.01 vs DMSO.

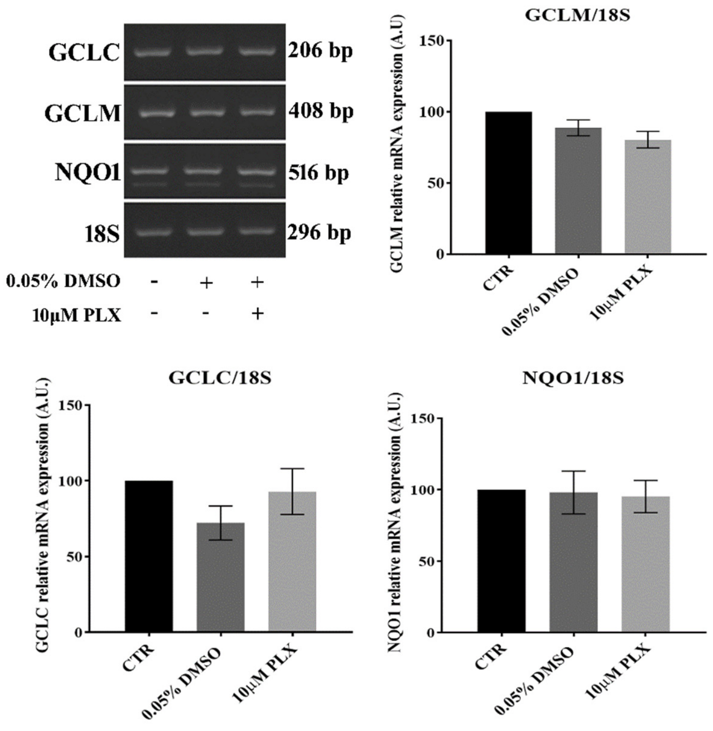

Supplementary Figure S2: NRF2 dependent gene analyses after 24h cell treatment with PLX4032. GCLM, GCLC and NQO1 mRNA expression was assessed by RT-PCR in MeOV-1 cells treated with 10μM PLX4032 for 24h. 18S mRNA was used as a housekeeping gene. Upper left panel: representative bands. Data denote mean ± S.E.M., n = 4 independent experiments.

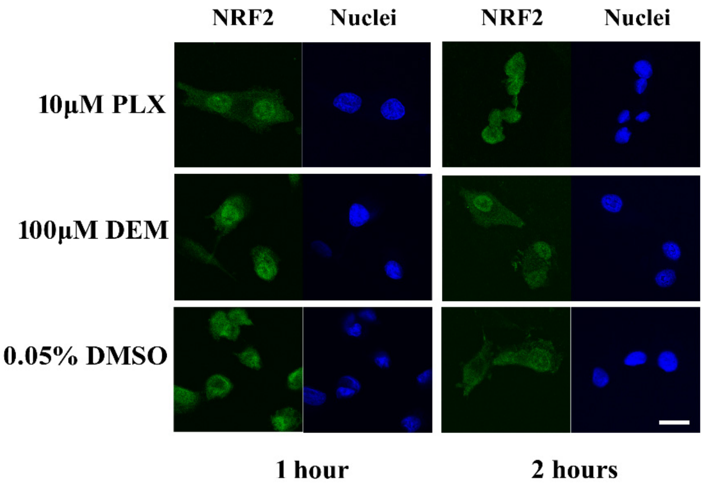

Supplementary Figure S3: NRF2 nuclear translocation after acute PLX4032 treatment. Panels show immunofluorescence for NRF2 in MeOV-1 cells treated with 10µM PLX4032 for 1h and 2h. Treatment of cells with 100µM diethylmaleate (DEM), known to activate NRF2, served as a positive control stressor. To-Pro3 was used to stain nuclei. Images are representative of 2 independent experiments and images were acquired using a 3-channel TCS SP2 laser-scanning confocal microscope (Leica Mycrosystem). Scale bar 20µm.
